# Effectiveness of Two Stress Reduction Interventions in Patients with Chronic Diabetic Foot Ulcers (PSY-DFU): Protocol for a Longitudinal RCT with a Nested Qualitative Study Involving Family Caregivers

**DOI:** 10.3390/ijerph19148556

**Published:** 2022-07-13

**Authors:** M. Graça Pereira, Margarida Vilaça, Eugenia Carvalho

**Affiliations:** 1 Psychology Research Centre (CIPsi), School of Psychology, University of Minho, 4710-057 Braga, Portugal; margaridavilaca@psi.uminho.pt; 2 Center for Neuroscience and Cell Biology, University of Coimbra, 3004-504 Coimbra, Portugal; ecarvalh@cnc.uc.pt; 3 Institute for Interdisciplinary Research (IIIUC), University of Coimbra, 3030-789 Coimbra, Portugal

**Keywords:** diabetic foot ulcer, clinical distress, muscular relaxation, hypnosis, physiological indicators of healing prognosis, quality of life, RCT, protocol study

## Abstract

Diabetic foot ulcer (DFU) is the leading cause of lower-limb amputations, with a significant impact on patients, families, and society. Since DFU medical treatments represent a major socioeconomic burden, cost-effective interventions are needed. This trial aims to assess the effectiveness of a muscle relaxation intervention compared to a hypnosis intervention versus active and passive control groups on DFU healing, physiological indicators of healing prognosis, and quality of life (QoL) in clinically distressed patients with a chronic DFU. A multicenter, randomized controlled trial with three assessment moments (baseline, two months post-intervention, and four months follow-up) will be conducted. Approximately 170 patients will be randomized and allocated to either treatment or control groups. Primary outcomes will be DFU healing, physiological indicators of healing prognosis, and QoL. Secondary outcomes will include perceived stress, psychological morbidity, and DFU representations. The efficacy of sessions on DFU healing will be qualitatively assessed in 12 patients allocated to the treatment and active control groups, as well as their family caregivers. This study will provide evidence regarding the effectiveness of two psychological interventions for the DFU healing process and the QoL of patients, with direct clinical relevance regarding DFU treatment and recurrence.

## 1. Background

Diabetes represents a growing public health concern with a growing global incidence during the last three decades [1] due to lifestyle changes and aging. According to the International Diabetics Federation, in 2021, an estimated 6.7 million adults have died as a result of diabetes or its complications. Over time, patients with diabetes have an increased risk of developing serious comorbidities, such as cardiovascular disease, blindness, kidney failure, and foot ulcerations [2].

Diabetic foot disease is one of the most serious complications of diabetes, impacting nearly 15% of all patients [3]. Diabetic foot ulcer (DFU) is a full-thickness wound below the ankle, in most cases, caused by poor glycemic control, calluses, ill-fitting footwear, underlying neuropathy, peripheral vascular disease, or improper foot care. Around 11–14% of worldwide patients diagnosed with diabetes will develop DFUs [4], which is the leading cause of lower-limb amputations in approximately 80% of these patients [5]. According to Zhang et al., the current DFU prevalence worldwide was 6.7%, while in Europe was 5.1% [6].

In addition to the devastating consequences of DFU development to patients and their families, the socioeconomic cost involved has become a burden. From 2007 to 2021, the direct cost of DFU treatment worldwide has increased from USD 232 to 966 billion, which reflects a dramatic increase. In Europe alone, the total medical costs for the management of DFUs in 2010 were estimated to be USD 105.5 billion, while in 2030, it is expected to reach USD 124.6 billion [2]. Thus, planning cost-effective interventions focused on DFU recovery becomes essential. This is also true regarding the impact on the environment, fewer trips to DFU clinics, fewer hospitalizations, and less clinical waste produced.

It is known that adherence to self-care behaviors and treatment is essential to wound healing. However, psychological factors, such as depression or anxiety, may also negatively influence DFU healing via psycho immunological effects [7,8]. Furthermore, DFU is associated with distress [9], and negative emotions contribute to prolonged infections, delayed wound healing, and poor quality of life (QoL), which is associated with low treatment responses and low remission rates, a major health concern [7,10,11].

Previous literature has emphasized the impact of stress on wound healing, almost exclusively in acute wounds, e.g., [12]. In fact, stress raises the level of cortisol, which has a negative impact on the immune system, particularly on wound repair due to immunity suppression [13,14]. By increasing the release of proinflammatory cytokines during tissue repair and driving tissue oxygen levels lower, psychological stress delays wound healing [15,16].

Stress may also impact the expression and function of microRNAs (miRNAs), important regulatory molecules that can be used as biomarkers for diagnosis or progression of complications from diabetes due to their ability to fine-tune cellular responses [17]. Thus, by modulating miRNA biogenesis, expression, and complex activity, stress can cause important changes in metabolism that may hamper DFU healing [18].

Glycemic control is one of the main strategies to monitor diabetes, reducing the complication of diabetes and the risk of hypoglycemia. Hemoglobin A1c (HbA1c) testing represents the best method to monitor glycemia in patients with diabetes, as it reflects levels of blood glucose over several weeks. Although distress has been associated with poor glycemic outcomes [19], the role of HbA1c in wound healing is not consensual. In fact, if some studies show direct associations between HbA1c levels and wound-healing rate, e.g., [20], other studies found no association between this biomarker and wound outcomes, e.g., [21].

Since there is evidence that psychological distress affects negatively wound healing [11], it is expected that reducing-stress interventions have positive implications on DFU recovery. Adjuvant interventions as relaxation training techniques have shown promising results in patients with diabetes [22,23] and patients with chronic DFUs [24,25]. Previous research also indicated hypnosis as an effective adjunct treatment in the management of diabetes, contributing to reduced blood glucose levels, better metabolic control, and increased blood flow to extremities, decreasing the risk of diabetic foot problems [26,27,28,29].

Although there is evidence suggesting the benefits of psychological interventions to patients with DFUs, the effectiveness of stress reduction interventions in DFU recovery, physiological indicators of wound healing, and patients’ QoL improvement has not been established. This is mainly because there are limited data evaluating the efficacy of psychological treatments in this area of research [30]. Thus, randomized controlled trials of low-cost psychological interventions to focus on the promotion of QoL and DFU healing are required. This paper describes the development of the PSY-DFU study protocol, which focuses on two interventions to reduce and manage stress and summarizes the advantages and limitations of this fully scripted treatment approach.

## 2. Methods

### 2.1. Objectives

The present study aims to:

1. Assess the effectiveness of a muscle relaxation intervention with guided imagery (TG1) compared to hypnosis with guided imagery (TG2) versus a neutral guided imagery placebo (ACG) and a group that does not receive any psychological intervention (PCG) regarding DFU healing, physiological indicators of healing prognosis, and QoL in patients with clinical distress and a chronic DFU.
2. Understand the perspectives of patients and family caregivers on the efficacy of TG1 and TG2 interventions versus ACG sessions for DFU healing.

#### Primary Specific Aims

The primary specific aims of this RCT are: (i) to compare the impact of both treatment groups (TG1 and TG2) in regard to DFU healing, physiological indicators of healing prognosis (biochemical parameters, inflammatory and angiogenic markers, miRNAs, and immune cells), and QoL, compared to control groups; (ii) to compare patients pre and post-intervention in the TG1 and TG2, controlling for patients’ health literacy, clinical characteristics (e.g., duration of diabetes and DFU, type of diabetic foot), and sociodemographic variables (e.g., gender, age, education, socioeconomic level); and (iii) to compare the efficacy of TG1 and TG2 versus ACG to DFU healing, according to participants and their respective family caregiver’s perceptions.

### 2.2. Study Design

The current study is designed as a longitudinal, participant-blinded, sham-controlled, cluster randomized controlled trial (RCT) with a nested qualitative evaluation. The RCT study includes three-assessment periods over the course of six months, while the nested qualitative study involves an additional semi-structured interview two weeks after completing the treatment or placebo sessions.

**Figure 1:**
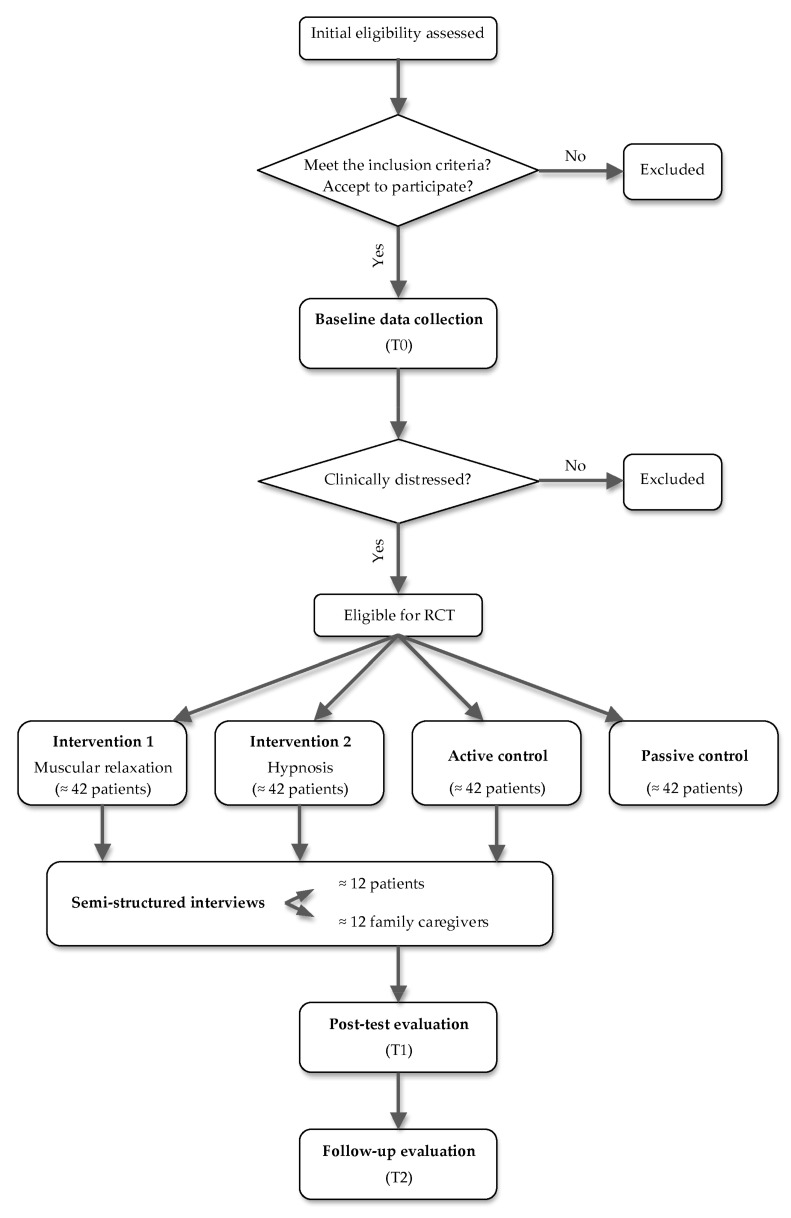
Study design and CONSORT flow chart.

The protocol for this RCT is based on the Standard Protocol Items: Recommendations for Interventional Trials (SPIRIT) 2013 Checklist [31] (Table 1). The study design and flowchart of the protocol, following the CONSORT 2010 standards [32], are summarized in Figure 1. The study will be conducted in three major hospitals in the North of Portugal with multidisciplinary diabetic foot consultations, and was approved by the Ethics Committee of the three hospitals. The study is also registered on the ClinicalTrials.gov platform since the 7th of January 2021 (Registration number: NCT04698720).

### 2.3. Participant Recruitment and Selection Criteria

Patients with chronic DFU attending the first consultation of the multidisciplinary outpatient clinical of diabetic foot ulcer from three major hospitals in Northern Portugal will be recruited. In the first consultation, researchers (Researchers 1, 2, and 3) will collect the clinical information of each patient to signalize those that meet eligibility criteria. Patients will be consecutively enrolled in the study.

The inclusion criteria are: (i) being 18 years old or more, (ii) having one or two diabetic chronic ulcers (a non-healing ulcer for six or more weeks and less than 12 weeks; in case of patients with two active chronic DFUs, the ulcer with the largest area will be selected as the index ulcer) at the time of baseline assessment, (iii) reporting clinical levels of psychological distress, and iv) providing written informed consent. Clinical distress is assessed according to the Hospital Anxiety and Depression Scale (HADS) [33] and the Perceived Stress Scale (PSS) [34], with patients scoring ≥11 on the HADS subscales or ≥13 (male patients) and ≥17 (female patients) on the PSS evaluated as being clinically distressed.

Exclusion criteria include: (i) the DFU, at the time of baseline assessment, being a relapse; (ii) having three or more DFUs at the time of baseline assessment; (iii) having undergone a transplant; (iv) being on hemodialysis treatment; (v) having a cancer disease; (vi) having degenerative dementia or severe psychiatric illness (e.g., schizophrenia), (vii) receiving psychological counseling, or (viii) taking psychiatric medication during the study period.

For the qualitative study, participants that completed at least 75% of the intervention sessions or placebo sessions (active control group) will be invited to participate, together with their family caregiver. Participants will be purposively selected according to the type of diabetic foot (neuropathic versus neuroischemic) and DFU progression (positive versus negative). The sample will also be selected according to participants’ capacity to provide in-depth and rich-textures information regarding the intervention’s effectiveness since qualitative purposive sampling is considered to be more efficient than random sampling [35]. Thus, the quantitative study sample will include four single-per-participant interviews per group (4 conditions × 3 groups), in a total of 12 patients’ interviews and 12 caregivers’ interviews.

### 2.4. Randomization

All eligible patients will be randomly assigned to one of the four groups—TG1, TG2, ACG, or PCG—in varying size blocks so that, over time, all groups can have a similar number of participants [36,37]. An independent researcher (Researcher 4), unaware of the numeric coding for each group, will create the randomization sequence using an online random number generator (https://www.graphpad.com/quickcalcs/randomize1/, accessed on 15 December 2021) with a 1:1 allocation, using random block sizes of 12. Researchers 2, 3, and 4 are blind to the block size used in the randomization procedure.

A priori stratification was defined considering the three hospitals where data collection will take place, as well as two common comorbid conditions deemed as factors of poor prognostic outcomes in patients with DFUs, specifically chronic kidney disease (CKD) and peripheral arterial disease (PAD) [38,39], to ensure that distribution of patients between groups is balanced in terms of confounders. Thus, randomization is stratified according to (i) hospitals; (ii) CKD and its disease stages (without CKD/CKD stage 1 and 2/CKD stage 3 and 4); and (iii) PAD (without PAD/with PAD), in a total of 12 possible strata.

Participants’ blinding will be maintained throughout the conduct of the trial, whereas Researchers 1, 2, and 3 will be aware of the allocation since they are responsible and will conduct the TG1 and ACG sessions. Quantitative data analysts will be blinded to participant allocation. The randomization process will be conducted at the end of each week to reduce the time interval between the randomization and the beginning of interventions.

## 3. Outcomes and Measures

### 3.1. Participant Timeline

Participants in the intervention and control groups will be assessed at the pre-intervention baseline (T0), at the end of the intervention/two months later at post-test (T1), and six months after T1 at the follow-up (T2). Data will be collected through face-to-face interviews conducted by health psychologists (Researchers 1, 2, and 3). Regarding the biochemical sample collection (biochemical parameter, inflammatory and angiogenic markers, miRNAs, and immune cells), a blood sample will be collected during the consultation at the T0, T1, and T2 assessment moments (Table 1).

### 3.2. Characteristics of Patients

Sociodemographic and clinical characteristics of all participants will be assessed.

#### 3.2.1. Sociodemographic Characteristics

Sociodemographic data will be collected prior to intervention through the Sociodemographic Questionnaire, a questionnaire developed for the purposes of this study that includes variables such as gender, age, education, residence, marital and professional status, having (or not) an informal caregiver, socioeconomic level, smoking history, and alcohol intake, among others.

#### 3.2.2. Clinical Information

Clinical data will be collected through the Clinical Questionnaire, developed specifically for this study, to be completed by the patients’ physician or nurse in the three assessment moments. This questionnaire asks about the type and duration of diabetes, complications associated with diabetes, diabetic foot type, DFU location and duration, concomitant treatment, ulcer healing time, and new DFUs. The clinical questionnaire also includes the Perfusion, Extent, Depth, Infection, and Sensation (PEDIS) classification system, proposed by the International Working Group of the Diabetic Foot, thus gathering information regarding the five most important categories to evaluate the DFU stage and progression [40].

### 3.3. Measures of Primary Outcomes

Primary outcomes are DFU healing, physiological indicators of healing prognosis, DFU impact on QoL, and physical and mental QoL.

#### 3.3.1. Degree of DFU Healing

Wound healing is defined as the complete epithelization of the wound and is assessed through the RESVECH 2.0-PT [41], a useful and validated tool to monitor the progression of chronic wounds of any etiology through six main parameters: wound area, depth and involved tissues, wound margins, type of tissue in the wound bed, levels of exudates, and presence of signs of infection/inflammations. This questionnaire will be filled out by the participant’s physician or nurse at the end of consultations to monitor DFU progression. Scores range from 0 to 35, where zero indicates complete healing and 35 corresponds to the worse possible wound.

#### 3.3.2. Physiological Indicators of Healing Prognosis

Physiological indicators include a biochemical parameter (HbA1c), inflammatory (IL-6, TNF-α, lymphocyte populations) and angiogenic (VEGF) markers, miRNAs (miRNA-21, miRNA-155), and immune cells (lymphocytes T effective and naïve). The quantification of HbA1c in the plasma will be performed using the competitive inhibition enzyme immunoassay Cloud Clone Corp. The quantification of the plasma inflammatory cytokines levels (IL-6) and tumor necrosis factor (TNF-α) will be performed using a LEGENDplex™ Human Angiogenesis Panel 1 Mix and Match (9-plex). Blood lymphocyte populations will be assessed in the whole blood by flow cytometry and an automated hematological cell counter. Levels of VEGF will be assessed through the serum by ELISA from Cell Signaling Technology. miRNA-21 and miRNA-155 will be assessed through blood collected in a tube from Qiagen (PAXgene Blood RNA Tubes, Qiagen, Hombrechtikon, Switzerland), and subsequently, the RNA will be converted into cDNA by “miScript Reverse Transcription Kit” (Qiagen). Amplification of miR-21 will be done through the light Cycler system with SYBR Green PCR Kit. The control gene will be the RNU6B. Immune cells will be calculated through the ratio of lymphocyte effectors CD4/CD8 to lymphocyte naïve CD4/CD8 cells. Blood samples will be collected and processed in the clinical laboratory of two of the three hospitals where data collection will take place.

#### 3.3.3. Impact of the DFU on QoL

The impact of the DFU on patients’ QoL will be assessed through the Diabetic Foot Ulcer Scale-Short Form (DFS-SF) [42], a self-report questionnaire to be fulfilled by participants to evaluate the effect of the DFU on patients’ general QoL.

#### 3.3.4. Physical and Mental QoL

Patients’ physical and mental health-related QoL will be assessed through the Short-Form Health Survey (SF-36) [43], a self-report questionnaire administered to participants.

### 3.4. Measures of Secondary Outcomes

Data will also be used to assess the following secondary outcomes: perceived stress, psychological morbidity, and DFU representations.

#### 3.4.1. Perceived Stress

The overall stress perceived by patients will be assessed through the Perceived Stress Scale (PSS) [34], a self-reported measure to be answered by participants.

#### 3.4.2. Psychological Morbidity

Psychological morbidity or emotional distress will be assessed through the total score of the Hospital Anxiety and Depression Scale (HADS) [33], comprising both anxiety and depression subscales. HADS is also answered by participants.

#### 3.4.3. DFU Representations

Patient representations regarding the DFU will be evaluated through the Illness Perception Questionnaire—Brief (IPQ-B) [44], a self-report scale administered to participants.

### 3.5. Other Outcome Measures

Health literacy, blood pressure, heart rate, time to complete wound healing, and time to favorable healing prognosis will also be assessed.

#### 3.5.1. Health Literacy

Personal health literacy is evaluated through the Medical Term Recognition Test (METER) [45], a widely used health literacy self-assessment measure administered to participants.

#### 3.5.2. Blood Pressure

Systolic and diastolic pressure, in millimeters of mercury (mmHg), will be assessed through a validated and certified blood pressure measuring device.

#### 3.5.3. Heart Rate

Heart rate, in beats per minute (bpm), will be assessed through a validated and certified blood pressure measuring device.

#### 3.5.4. Time to Complete Wound Healing

This variable corresponds to the time interval (days) between the baseline assessment and the complete DFU healing.

#### 3.5.5. Time to Favorable Healing Prognosis

Based on the wound area, the time to a favorable prognosis will be assessed through the DFU area evolution (reduced versus increased, at the end of the participation in the study, i.e., T1 or T2 assessment moments).

### 3.6. Qualitative Assessment

Approximately 12 patient participants allocated to the TGs and ACG, together with their respective family caregivers, will be invited to take part in semi-structured interviews to share their perceptions on the efficacy of sessions for DFU healing. The interview guide consists of open-ended questions to be administered individually to patients and the family caregiver indicated by the patient. The script will be unchanged throughout the interviews. All interviews will be audio-recorded in digital audio and transcribed verbatim. The transcribed interviews will be anonymized to safeguard the confidentiality of data and participants.

Qualitative analysis of the interviews will be performed shortly after the transcription of the interviews. Interview contents will be inductively coded and theoretically analyzed through the thematic content analysis technique and later compared to the existing literature. To process, organize, code, and thematically analyze data, the NVivo software (QSR International PtyLtd, Melbourne, Australia) will be used. Researchers 1, 2, and 3 will be responsible for the interview implementation and the data analysis.

## 4. Interventions

### 4.1. Trial Arms

All participants will receive standard of care medical/nursing treatment for DFU, according to the guidelines of the Portuguese General Health Direction [46] and the International Working Group on the Diabetic Foot [47].

#### 4.1.1. Intervention Groups

Participants in the intervention groups are allocated in TG1 or TG2. Participants allocated to TG1 will receive four individual sessions of muscle relaxation with guided imagery (MR + GI), a technique of alternately tensing and relaxing muscle groups individually throughout the body. Relaxation intervention begins with diaphragmatic breathing, followed by Jacobson’s progressive muscle relaxation, which involves the contraction and subsequent relaxation of the 16 muscle groups of the body (hand, forearm, biceps, upper forehead, eye, nose, mouth, jaw and throat, neck, shoulder, chest, stomach, thigh, leg, and foot). The contraction will be performed for seven seconds while the relaxation lasts for about 40–50 s. The relaxation of the foot muscle group will not be performed on the foot with the wound because dressing and bandages may restrict the foot movements and, together with the typical joint stiffness of the diabetic foot, will render difficult the performance of exercise. After the muscle relaxation exercises, the guided imagery focused on the DFU healing process will initiate. The participant will be instructed to think about his/her current state of health and to imagine the DFU as a decreasing dark area and the healing process as a light associated with pleasant sensations. After being trained by the Lead Researcher, Researcher 1, 2, or 3 will be responsible for conducting the TG1 sessions in the respective hospitals.

In the TG2, participants will benefit from four individual sessions of hypnosis with guided imagery (H + GI), conducted by three qualified hypnotherapists external to the research study. The first session begins with the Eye-Roll Test for Hypnotizability [48]. Each session follows the Hypnotic Protocol with the following steps: pre-talk/absorption/ratification/alitiation/dissociation/awakening. All hypnotic sessions will train participants in the visual, auditory, and kinesthetic perception of ulcer healing, and will also promote medical treatment adherence.

The protocol for both treatment groups includes four scripted sessions, each one with duration of approximately 45 min, delivered once every two weeks, resulting in approximately a 2-month treatment course. Sessions will be conducted in a consulting room reserved by the hospital for the study, where patients will lie on a specialist couch.

#### 4.1.2. Control Groups

Participants in the ACG will receive neutral guided imagery sessions, i.e., sessions focused on the patient’s daily life before the DFU. Neutral sessions are conducted by Researchers 1, 2, and 3 following a scripted protocol that includes four biweekly sessions of approximately 45 min, each one dedicated to a different topic (family, work, friends, and leisure). Initially, the participant will be asked to choose a specific event related to the topic of the session, positive or negative, without telling the researcher which event he/she thought about. Then, the participant will answer several questions regarding the episode to promote a more detailed reconstruction of the event, being instructed to only think about the answers and not to reply orally. When the whole episode is remembered, the participant will be asked to tell what he/she imagined/remembered regarding each of the questions. The goal of the placebo sessions is to control for the effect of the received attention, from psychologists and hypnotherapists, on patients in the treatment groups. It is possible that the privileged contact may positively influence the patient’s healing process. Therefore, the attention control condition will allow us to differentiate between the impact of interventions versus the attention given to the ACG.

Participants in the PCG will not receive any intervention besides the standard of care medical/nurse treatment. Both passive and active control patients will complete the same outcome questionnaires as the participants allocated to the interventions, at the same time intervals.

### 4.2. Adherence to the Treatment Plan

Patients will be fully informed about the study goals, procedures, and potential benefits that may arise from the results of the DFU treatment. Assessment moments, intervention, and placebo sessions will be scheduled for diabetic foot appointment days so that patients will not have to go to the hospital on purpose to participate in the study. Before the appointment day, Researchers 1, 2, and 3 will call patients to remind them of their appointment with the research team for the purpose of the study. Participants will be asked to complete the last assessment (T2) even if they miss the mid-term assessment (T1).

To engage the health professional staff (e.g., physicians, nurses, podologists), meetings will be held with the physicians responsible for the multidisciplinary consultation of diabetic foot and head nurses (in the three hospitals involved in the study), in order to introduce the study and clarify any questions that may arise.

## 5. Statistical Analysis

### 5.1. Sample Size

Using Sakpal’s formula [49] and according to the descriptive results of the pilot study [50], considering the difference in the mean (1.93) and standard deviation (6) of the treatment versus passive control groups, with a statistical power of 80% and a statistical significance level of 5%, a definitive RCT will require 152 participants. Considering a dropout rate for intervention sessions of 11%, a definitive RCT with four groups will require a sample size of 169 participants, with 42 patients per group [49].

### 5.2. Data Analysis

The baseline data of the treatments and control groups will be compared using the chi-squared test for binary variables and the independent sample t-test for continuous variables. To prevent overestimating the impact of both interventions, an intention-to-treat approach will be assumed to evaluate the impact on the primary and secondary outcomes. The comparison between the treatment groups versus control groups over time (differences between and within) will be performed through mixed general linear models of analysis of variance for repeated measures. Effect sizes (Cohen’s d coefficient) will also be provided between T0 and T1, T1 and T2, and T0 and T2 to determine if the intervention shows any treatment effect. A significance level of 0.05 will be set for a two-sided test, and the 95% confidence interval will be calculated. In addition, time to complete wound healing and time to favorable healing prognosis (based on the wound size) will be estimated using survival analysis, specifically, single variable and multivariable Cox proportional hazard ratio regression models and Kaplan–Meier plots. All previous analyses will include the DFU stage at the baseline as a control variable. Other confounding clinical factors associated with the outcome variables will also be considered in the statistical analysis.

The data will be analyzed using the Rstudio, R version 3.6.2 (R Core Team, Vienna, Austria) and the SPSS statistics, v. 24.0 (IBM Corp., Armonk, New York, NY, USA).

## 6. Ethics

Only patients that provide written consent to participate in the trial and sign a consent form (to allow access to their medical records) will be included in the study. The consent process continues throughout the study as researchers review study procedures and confirm patient’s wish to continue at each session and assessment moment. All participants will be aware that they are free to withdraw from the trial at any time without any effect on their future standard of care medical treatment. For the qualitative nested study, patients and caregivers will receive a specific written informed consent to be signed.

The personal data of participants will be kept confidential before and during the study by pseudonymization, with the participant’s name being coded. Names and other identifying information will be kept on a single page in the database, only accessible to Researcher 1. At the end of the study, participant anonymization will be fully guaranteed. All personal data collected within the framework of this study will be preserved until the final publication of the results, not exceeding a five-year period. After this period, all data will be destroyed (paper records and biochemical samples) or eliminated (database records).

## 7. Discussions

Given the huge challenge of distress in patients with DFUs, and the limited data evaluating the healing process and QoL of patients with chronic DFUs, the study of the efficacy of two economic stress reduction interventions in clinically distressed patients with chronic DFUs is proposed.

This paper describes the PSY-DFU study protocol, the first national and international multicenter study to address the contribution of psychological variables in DFU healing and QoL. The study also tests the effects of two psychological interventions: individual relaxation and hypnosis, both with guided imagery, on wound healing, physiological indicators of wound healing prognosis, and QoL.

Many complicating factors of diabetes compromise the healing process, making effective treatments challenging. The compliance rate in the treatment groups is one of the major difficulties experienced so far. This is, in part, due to the fact that patients with chronic DFUs often present a long history of medical care, and yet many fail to adhere to the medical treatment and self-management, thus developing negative expectations about psychological interventions. Moreover, in the treatment groups, when DFU is completed healed, the intervention ends, which also contributes to the low-rate compliance. Regarding the DFU progress monitoring, this study would benefit from more advanced and accurate techniques to assess wounds, such as imaging devices. However, none of the three participating hospitals have yet adopted imaging systems to routinely measure DFUs. In addition to the limited strategies to improve adherence to treatments, and the use of more traditional tools to measure chronic DFUs, the non-fully blinded design also represents a limitation of this research.

The multicenter and randomized design of this PSY-DFU study, together with the used mixed-methods approach, is one of its major strengths. Results will answer the question, “Which psychological intervention is more effective and has more impact on the primary and secondary study outcomes in clinically stressed patients?” The findings will help to clarify the mechanistic underpinnings of the relationship between distress and chronic wound healing, the knowledge that is still very limited in general and nonexistent in DFU. The RCT is enhanced by the qualitative nested study, as qualitative data are expected to refine findings regarding the effectiveness of each intervention, allowing a better understanding of the impact of psychological factors on the DFU healing process.

Finding psychological and biological clues from specific patient groups with chronic DFUs will help health professionals tailor better treatments with precision that could prevent future diabetic foot wounds from developing or from recurring. Specifically, identifying the specific risk profile of patients will best provide health professionals with important information regarding their psychological vulnerabilities, which may make it difficult for DFU healing. Additionally, survival analysis will help to identify the best assessment moments to initiate psychological interventions to contribute to the DFU healing, and prevent the onset of new wounds. Therefore, this study is of important clinical relevance regarding DFU healing and recurrence, as well as QoL, and may also contribute to the decrease in health care and medical-related costs associated with DFU treatments, including amputations.

## 8. Conclusions

The present study intends to contribute with scientific knowledge regarding the progression of chronic DFUs in distressed patients, as well as to test the effectiveness of two stress reduction interventions for DFU healing, including the perspectives of patients and family caregivers. Thus, this study will surely have direct clinical relevance regarding DFU treatment and recurrence, with a significant impact on the QoL of patients and their family caregivers.

## Figures and Tables

**Table 1:** Schedule of enrollment, interventions, and assessments of the PSY-DFU study, following the Standard Protocol Items Recommendations for Interventional Trials (SPIRIT) guidelines.

TIMEPOINTS	Enrollment	Allocation	Pos-Allocation			Follow-Up
		T0	T0	T1	2 Weeks after T1	T2
Eligibility	X					
Informed consent	X					
Allocation		X				
INTERVENTIONS			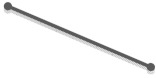			
PMR + GI (TG1)						
H + GI (TG2)						
Control groups			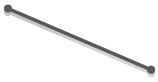			
Active (ACG)						
Passive (PCG)						
ASSESSMENTS						
Sociodemographic data			X			
Health literacy			X			
Clinical data (PEDIS)			X	X		X
DFU evolution/healing ^a^			X	X		X
Impact of DFU on QoL			X	X		X
Mental and physical QoL			X	X		X
Perceived stress			X	X		X
Psychological morbidity			X	X		X
DFU representations ^a^			X	X		X
Blood pressure ^b^			X	X		X
Heart rate ^b^			X	X		X
Biochemical parameter			X	X		X
Inflammatory markers			X	X		X
Angiogenic markers			X	X		X
miRNAs			X	X		X
Immune cells			X	X		X
Semi-structured interviews					X	

QoL—quality of life; PMR + GI—progressive muscular relaxation with guided imagery; H + GI—hypnosis with guided imagery; ACG—active control group; PCG—passive control group; PEDIS—Perfusion, Extent, Depth, Infection, and Sensation; DFU—diabetic foot ulcer. ^a^ In T1 and T2, when the DFU is healed, the DFU progression and DFU representations are not assessed. ^b^ Blood pressure and heart rate are assessed before and after each intervention (PMR + GI and H + GI) and placebo (ACG) session.

## Data Availability

Not applicable.
